# Combining Micro-Raman Spectroscopy and Scanning Electron Microscopy Mapping: A Stony Meteorite Study

**DOI:** 10.3390/ma14247585

**Published:** 2021-12-10

**Authors:** Maya Musa, Riccardo Rossini, Daniela Di Martino, Maria Pia Riccardi, Massimiliano Clemenza, Giuseppe Gorini

**Affiliations:** 1 Department of Earth and Environmental Sciences, University of Pavia, 27100 Pavia, Italy; maya.musa@unipv.it (M.M.); mariapia.riccardi@unipv.it (M.P.R.); 2 Physics Department and INFN Section, University of Milan Bicocca, 20126 Milan, Italy; riccardo.rossini@unimib.it (R.R.); massimiliano.clemenza@unimib.it (M.C.); giuseppe.gorini@unimib.it (G.G.)

**Keywords:** micro-raman spectroscopy, mapping, scanning electron microscopy with energy dispersive spectroscopy, meteorite, chondrite, chondrule, thin section

## Abstract

Meteorite characterisation represents a privileged and unique opportunity to increase our knowledge about the materials composing the Universe and, particularly, the Proto Solar System. Moreover, meteorites studies evolve contextually with the development of analytical technologies. In the present paper, the results from an unclassified stony meteorite (chondrite) characterisation have been reported on the basis of the innovative analytical protocol presented here. Advanced Mapping by micro-Raman Spectroscopy and Scanning Electron Microscopy equipped with Energy Dispersive Spectroscopy have been combined to disclose molecular and elemental features on the same regions sample at a micrometric resolution. Thanks to their non-destructive properties, the mapping tools of both instruments have been applied to single chondrules analysis and the best match between the mineralogical information and the chemical composition has been obtained. This combined approach proved to be highly suitable in disclosing the crystallinity features of the phases, with in-depth spatial and morphological details too.

## 1. Introduction

“Meteorite” is a generic term, used to identify all the objects falling from Space to the Earth, although a huge variability characterises these materials. In fact, the differences in the genesis of the asteroids or their parental bodies originating from the meteorites reflect their history (i.e., collisions, etc.), and are responsible for the meteorites’ heterogeneity [1,2]. These fascinating and rare materials play a fundamental role in the study of planetary and cosmological issues. Indeed, they represent a unique opportunity to study the elements and the materials constituting the proto-Solar System, including the Earth precursor [3,4,5].

The classification of meteorites is a very complex procedure and requires in-depth knowledge of the material, e.g., the internal chondrules composition [6,7,8].

Meteorites can be grouped into three main categories: stony, iron, and stony-iron meteorites. Stony meteorites consist of mostly silicate minerals and may contain small spheroidal grains (known as chondrules and containing mafic minerals) or other inclusions in the range size from sub-mm up to cm. The most common stony meteorites (ordinary chondrites) usually have an inhomogeneous phase and elemental distribution throughout their volume [7].

Raman spectroscopy is a well-established technique for identifying a wide range of substances and is an effective methodology for characterizing small particles and inhomogeneous solids. Raman technique is also particularly suitable to characterise the mineral phases [9], even if the phases are polymorphs or minerals belonging to the same series [10]; moreover, this analysis allows several considerations about the phases’ crystallinity [11]. Meteorite samples have also been studied [12,13,14], looking at mineral phases or the presence of carbon. Wang et al., (1999) [15] not only studied Martian meteorites using Raman but also suggested a Raman system to be used remotely on Mars for in situ analysis of minerals. More recently, several micro-Raman mapping systems have been developed, allowing the complete spatial resolved interpretation of the data. In few cases, this Raman mapping has been applied to study meteorites, and especially for the carbon-components identification [16,17,18].

On the other hand, Scanning Electron Microscopy (SEM) combined with an Energy Dispersive Microanalysis (EDS) System represents a technique traditionally applied to meteorite studies, thanks to the possibility of carrying out a comprehensive chemical characterisation. Thus, the two techniques appear particularly suitable for meteorite characterisation [19,20]. Moreover, as for the Raman mapping techniques, the SEM-EDS mapping tool has also been recently proposed for meteorite study [21].

By the combination of the two techniques, a sample—especially a polyphase system—can be completely characterised, from a mineralogical, chemical and morphological point of view, as previously demonstrated [22]. Thanks to the non-destructive characteristic of the techniques, a similar methodology is proposed here for meteorite characterisation, where the mapping tools of both techniques have been applied to the study, not only of the same meteorite, but exactly of the same chondrule, in order to obtain data perfectly matching and related to the morphology observed.

Finally, this work is intended as an initial methodology study in the framework of a comprehensive meteorite characterisation project, focused on non-destructive multidisciplinary analytical protocol route.

## 2. Materials and Methods

### 2.1. Material

**Figure 1:**
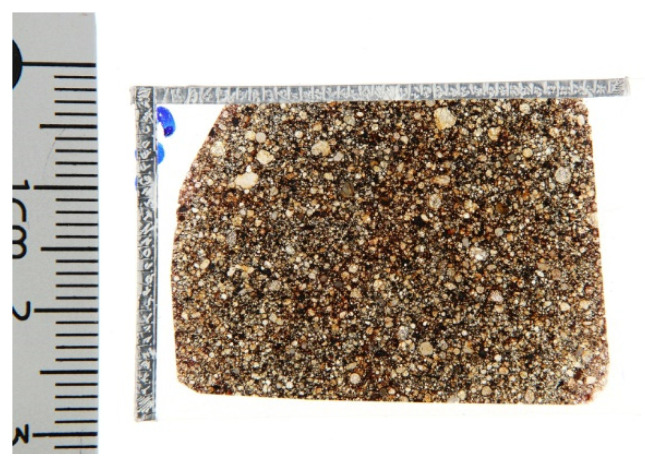
Image of the meteorite thin section used as study-case sample for the present study, in back-diffused light. Several chondrules structures are clearly visible at naked eye. The fixed coordination system made by Al-tape stuck on the supporting glass side is also visible.

The analysed sample consists of a standard petrological thin section [23], 30 µm thick, cut for material identification purposes from an unclassified meteorite of a private collection (see Figure 1). It must be underlined that the thin section has not been specifically prepared for the present study, but it has been used as a study case in order to test the multianalytical methodology developed, without requiring new material sampling. Regarding the meteorite, it has been identified as a stony meteorite and, more precisely, a chondrite, though it is still lacking a complete classification. The only information available was that the meteorite came from Middle East desert areas, and it was included more than 20 years ago in a private collection; therefore, a small fragment and the thin section from this last one has been cut in order to identify the sample as a meteorite from Space or a rock from Earth.

As shown in Figure 1, a fixed coordinate system has been stuck on the thin section, allowing precisely the same chondrules to be found easier and to be analysed by both techniques: micro-Raman Spectroscopy and SEM-EDS. After all micro-Raman Spectroscopy mapping analyses, a carbon coating was applied to increase the sample’s conductivity in order to carry out SEM-EDS investigations.

### 2.2. Micro-Raman Spectroscopy

The micro-Raman instrument used for the present work is a Renishaw In-Via Reflex μ-spectrometer (Renishaw Plc, Wotton-under-Edge, England, UK), endowed with two different laser sources: a 632.8 nm, 25 mW He-Ne laser and a 514.5 nm, 100 mW solid-state laser. The spectroscope detector consists of a Charge Coupled Device (CCD) and sets two different motorised (Synchroscan mode) gratings, 1800 l/mm and 2400 l/mm, respectively. The Raman spectroscope is coupled in confocality mode with a Leica optical microscope equipped (Leica Microsystems, Wetzlar, Germany), with polarising filters and different objectives: 3 Long Working Distance (LWD), 5× (0.12 Numerical Aperture-NA), 50× (0.75 NA), 100× (0.75 NA), and 2 Short Working Distance (SWD), 20× (0.40 NA) and 50× (0.50 NA).

The chondrule mapping analyses were performed on a xy grid of about half a chondrule, using a montage tool, focusing the 514.5 nm source (90% filtered, to avoid the eventual alteration of the unstable phases due to the laser energy) using a 20× objective and setting 10 µm as the spot step (reaching a spatial resolution of the order of microns). The Raman spectral region is between about 1200 and 200 cm^−^^1^ and is suitable for distinguishing, for example, silicate mineral phases, such as feldspar, olivine, and pyroxenes, and was investigated in static mode, centring the 2400 grating at 700 cm^−1^. In order to balance the signal against noise, 3 cycles of 5 s for each grid analysis spot were performed, for a total analysis time between about 24 h and 48 h for each map, depending on the chondrules dimension.

The correct calibration of the instrument was obtained by checking the position and intensity of the Si band at 520.6 ± 0.1 cm^−1^, focusing the laser on the internal standard sample before every map run.

The information obtained by the maps has been integrated by successive linear point to point longer acquisitions, performed by focusing the source by higher magnification objectives (100×) and/or exchanging the laser source in case of fluorescence.

The Renishaw’s software Wire vers. 5.5 has been used for system control, acquisitions, as well as map elaboration. In particular, to highlight the different mineral phases constituting the chondrules, the micro-Raman Maps have been elaborated by applying the intensity to baseline calculation tool.

### 2.3. SEM-EDS

Each chondrule characterised by micro-Raman spectroscopy was then studied by SEM-EDS analyses. X Ra Textural observations (backscattered electrons images—BSE) and micro-chemical compositions were performed by a Tescan FE-SEM, Mira 3XMU-series, Tescan Orsay Holding a.s., Brno, Czech Republic), equipped with an EDAX energy-dispersive spectrometer (Apollo XL silicon drift detector energy dispersive Xray—spectrometer—SDD-EDS). The operating conditions were: 20 kV accelerating voltage, 12 mA beam current, 15.8 mm working distance, counts of 100 s per analysis, and dead time of approximately 25%. The measurements were processed using the EDAX Genesis software and the data obtained using the ZAF correction. This method corrects for the effects that atomic number (Z), absorption (A), and fluorescence excitation (F) have on the intensity of the X-rays emitted and collected by the detector.

## 3. Results

Below we report the results obtained from the mapping analyses of two chondrules, representative of the methodology developed.

### 3.1. Chondrule A

**Figure 2:**
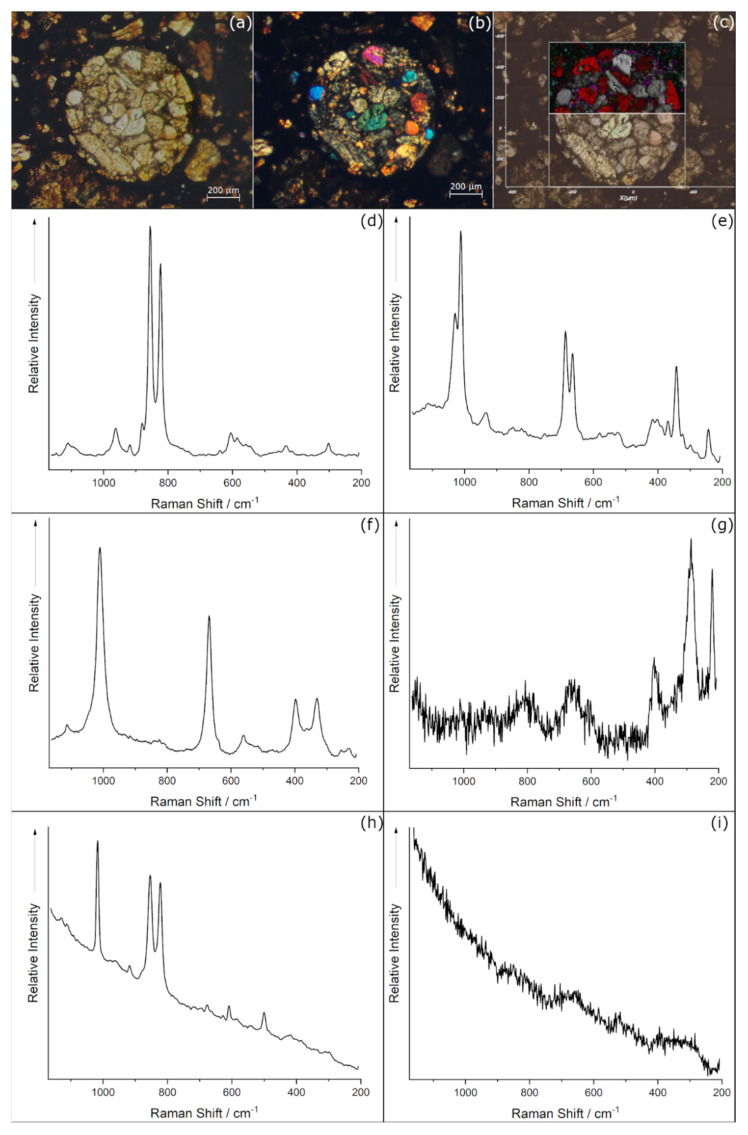
OM images and µ-RM of chondrule A: (**a**) OM image plane-polarized transmitted light; (**b**) OM image cross-polarised transmitted light; (**c**) µ-RM results overlapped on the OM image, the white thick square indicates the mapped area and the different colours correspond to the different mineral phases, identified on the basis of the Raman spectra: (**d**) Forsterite (grey), (**e**) Enstatite (red), (**f**) Diopside (purple), (**g**) Hematite (green), (**h**) Anhydrite (blue), and (**i**) Raman spectrum without any bands, corresponding to the black areas in the map.

In Figure 2, the combination of the Optical Microscope Images (OM) and micro-Raman Mapping (µ-RM) results has been reported.

A characteristic near round morphology of the chondrule, with a diameter of about 820 µm, with many phenocrystals of different shapes and dimensions, resulted in the OM image acquired in transmitted plane-polarised light (see Figure 2a). By transmitted cross-polarised light image (see Figure 2b), the typical intense interference colours of olivine and orthopyroxene phases [24] have been observed for the crystals, as expected for chondrules mineral composition [5,25]. On the other hand, the matrix surrounding the single phenocrystals and the chondrule appeared black, or rather opaque and/or without any optical birefringence activity.

For µ-RM on this chondrule, a total of 4217 spectra has been acquired. The map elaboration required a pre-check of all the spectra in order to identify the different mineral phases constituting the chondrule, also on the basis of the OM observations. The spectra corresponding to the mineral phases identified have been reported (see Figure 2d–i). In particular, the bands at 824 and 856 cm^−1^ (see Figure 2d) allowed the identification of the olivine as forsterite mineral phases [Mg_2_SiO_4_] [26], while two different pyroxene phases have been detected. In fact, the bands at1034–1013 cm^−1^ and 687–664 cm^−1^ (see Figure 2e) are ascribable to the enstatite pyroxene phases [Mg_2_(Si_2_O_6_)] while the bands at 1010 and 666 cm^−1^ (see Figure 2f) are consistent with diopside phase [CaMg(Si_2_O_6_)] [26,27]. Regarding the non-silicate phases, the bands at 220, 291 and 407 cm^−1^ (see Figure 2g) are characteristic of the hematite mineral phase [Fe_2_O_3_] [26,28] and the band at 1017 cm^−1^ (see Figure 2h) corresponds to the vibrational Raman modes of the anhydrite phase [CaSO_4_] [29]. It must be underlined that no bands corresponding to feldspar phases have been detected [30] as well as to ringwoodite mineral [31].

The main vibrational modes of the identified phases have been used to prepare the map and apply the signal to baseline calculation: the mapped ranges are [820; 830] cm^−^^1^ for forsterite, [682; 692] cm^−1^ for Enstatite, and [662; 672] cm^−1^ for diopside. It must be noted in the spectra (see Figure 2e,f) that the enstatite and diopside main bands fall in the same spectral region due to their structure similarities, and therefore the two maps partially overlap. Consequently, the choice of the mapping boundaries for these two phases has been performed heuristically, aiming to maximise the spatial distinction between them. For the non-silicate phases, the ranges are [1015; 1020] cm^−1^ and [285; 300] cm^−1^ to map the anhydrite and the hematite phases, respectively.

The µ-RM of the chondrule A has been reported in Figure 2c, where the spatial distribution of the different phases, recognisable by the different colours, have been overlapped to the OM image. By analysing the phases distribution, it is possible to note the differences in terms of the area occupancy and relative abundance. Within the two pyroxenes, the enstatite resulted in the dominant one, while the diopside appeared segregated in small areas, focused near margins of the enstatite crystals. The presence of non-silicates phases resulted in being relatively less abundant than the silicates: the oxides and the other phases appeared as crypto-crystals immersed in the matrix, and they were more present outside the chondrule diameter than in the area inside it. Finally, the black colour in the map corresponds to the sample areas where the spectra did not show any Raman bands, such as the one reported in Figure 2i.

**Figure 3:**
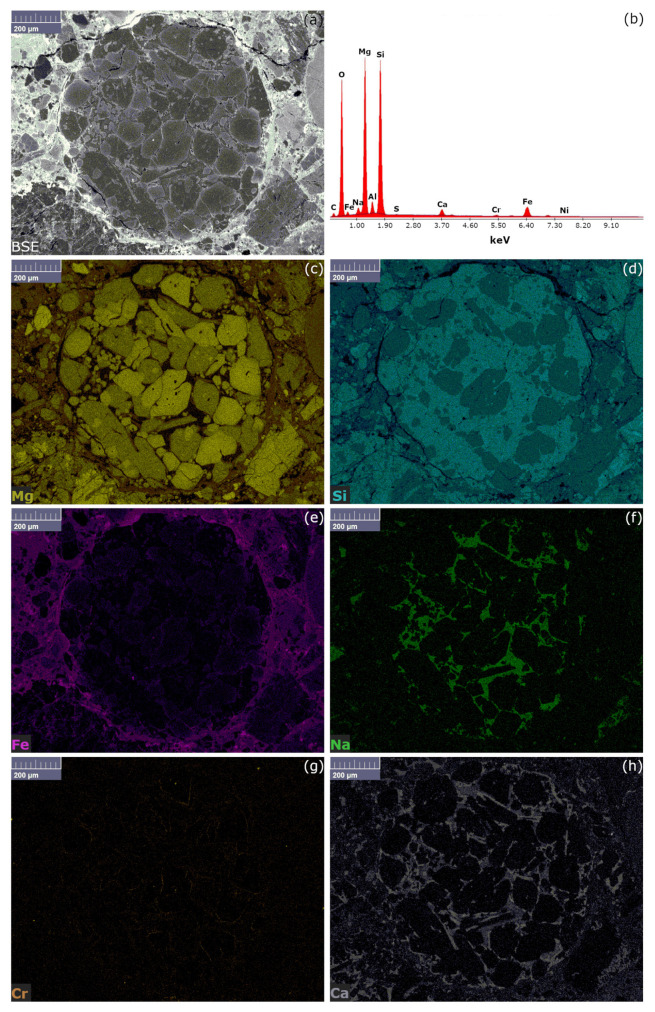
SEM-EDS results of chondrule A: (**a**) BSE image of the chondrule; (**b**) EDS spectrum corresponding to the sample’s area visible in (**a**); (**c**) Mg EDS map; (**d**) Si EDS map; (**e**) Fe EDS map; (**f**) Na EDS map; (**g**) Cr EDS map, (**h**) Ca EDS map.

The same chondrule studied by µ-RM, has been analysed by SEM-EDS, applying the mapping tool (EM) and results have been reported in Figure 3. Thanks to the application of the BSE detector, a preliminary chemical information spatial resolved has been carried out; indeed, the areas corresponding to a lower Z number appeared darker than the areas characterised by a higher Z number and, as shown in Figure 3a, the chondrule appeared darker than the areas surrounding it. Using EDS analysis (see Figure 3b), the interesting resulting area was mainly composed of silicon, magnesium, less iron, sodium, and calcium, with traces of chromium, nickel, and sulphur. By the elemental maps (see Figure 3c–h) it has been possible to observe the different elements distribution, mostly in agreement with the Raman results: Mg characterises the chondrule phenocrystals, previously identified as forsterite and enstatite, while Fe is concentrated mainly outside the chondrule. Fe was also present inside the chondrule, but only as a trace between the chondrules crystal and in the olivine chemistry. It is worth noting that Si has a ubiquitous distribution while Na and Ca are restricted in the matrix areas, surrounding as a rime the forsterite and enstatite crystals (see Figure 3f,h). Finally, Cr presented several similarities in Ca and Fe distributions regarding the inner and outer chondrule concentration, respectively (see Figure 3g). Ni and S have not been shown by EM, due to their extremely low concentration and spatial distribution.

**Figure 4:**
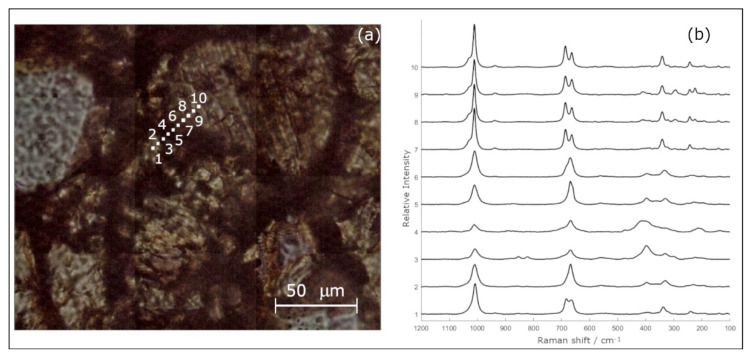
Line spots Raman mapping: (**a**) OM plane-Polarised Transmitted Light acquired by 100× objective in montage mode, where the position of the 10 spots analyses have been reported; (**b**) the 10 Raman spectra corresponding to the spots indicated in (**a**). A step of 5 µm between the different spots has been set to perform the line mapping. The spectra acquired on 1, 7, 8, 9, and 10 spots correspond to the enstatite phase, while the analyses carried out from the rime (2, 5, and 6) show bands compatible with the diopside phase. Finally, the spectra from the matrix (3 and 4) show the spectral features of the diopside plus broad bands centred around 400 and 220 cm^−1^. It must be also noted the absence of any doublet bands at about 475–510 cm^−1^.

The presence of Si-Na elements observed by EM, corresponding to black areas observed by Raman mapping, has been investigated deeper by a second µ-RM, in this case performed by point to point in line mode (see Figure 4). Ten spot analyses have been carried out, focusing the laser source by 100× objective, along a virtual line preset by the analyst between two different enstatite grains of chondrule A: from the inner part of the crystal through rime and matrix, to another enstatite. The spectra acquired on 1, 7, 8, 9, and 10 spots correspond to the enstatite phase, while the analyses carried out from the rime (2, 5, and 6) show bands compatible with the diopside phase. Finally, the spectra from the matrix (3 and 4) show the spectral features of the diopside plus other vibrational modes, such as broad bands in the lower part of the spectra. Those bands, cantered around 400 and 220 cm^−1^, can be correlated with the sulphide phases [32] and/or iron oxides in a low crystallinity state [22]. It must also be noted the absence of any doublet bands at about 470–510 cm^−1^, characteristic of feldspar phases [26,30].

### 3.2. Chondrule B

**Figure 5:**
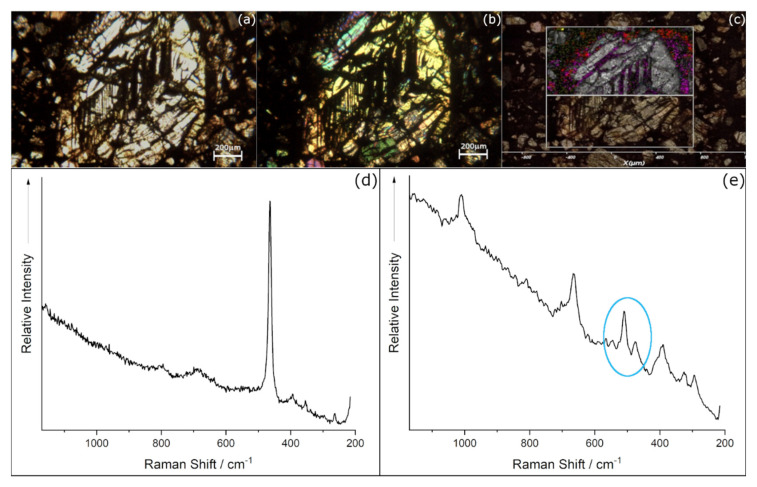
OM images and µ-RM of chondrule B: (**a**) OM image plane-polarized transmitted light; (**b**) OM image cross-polarised transmitted light; (**c**) µ-RM results overlapped on the OM image, the white thick square indicates the mapped area and the different colours correspond to the different mineral phases, identified on the basis of the Raman Bands spectra: Forsterite (grey), Enstatite (red), Diopside (purple), Hematite (green), Anhydrite (blue), (**d**) Quartz (yellow) and Raman spectrum without any bands, corresponding to the black areas; (**e**) µ-RM spectrum observed in some areas at the rime of the diopside crystal, the circled bands at 476 and 510 cm^−1^ are consistent with feldspar phase, while the others are ascribable to diopside.

In Figure 5, the combination of the OM images and µ-RM results has been reported. In particular, as shown by transmitted plane-polarised and crossed-polarised images (see Figure 5a,b), the chondrule B shows bigger dimensions (about 1200 µm) than chondrule A, and a particular texture, where the olivines are arranged in barred structures [33].

For µ-RM on this second chondrule, a total of 10,070 spectra were acquired. The identification procedure revealed mainly the same phases already found for chondrule A: forsterite for olivines, enstatite, and diopside for pyroxenes, plus anhydrite as non-silicate phase; thus, the same vibrational modes have been used to create the map (see Figure 5c), where the phases have been reported by the same colour code used for chondrule A. Unlike the chondrule A, the forsterite appears concentrated in the inner part of the chondrule, while the enstatite is segregated to the external areas, and the diopside, as previously shown, is present only as a rime of the grain or spot in the matrix. In addition, several more phases have been found for silicates: the quartz, mapped on the basis of its main vibrational mode at 464 cm^−1^ [26] (see Figure 5d) and feldspar, identified by the doublet at about 476 and 510 cm^−1^ [26,30]. In this second case, the presence also of diopside bands in the same spectra have been noted. It must be underlined that no bands corresponding to ringwoodite [31] have been detected.

Finally, the areas corresponding to spectra without bands or showing a broad scattering have been reported; those areas matched with the black ones visible at MO (see Figure 5b).

**Figure 6:**
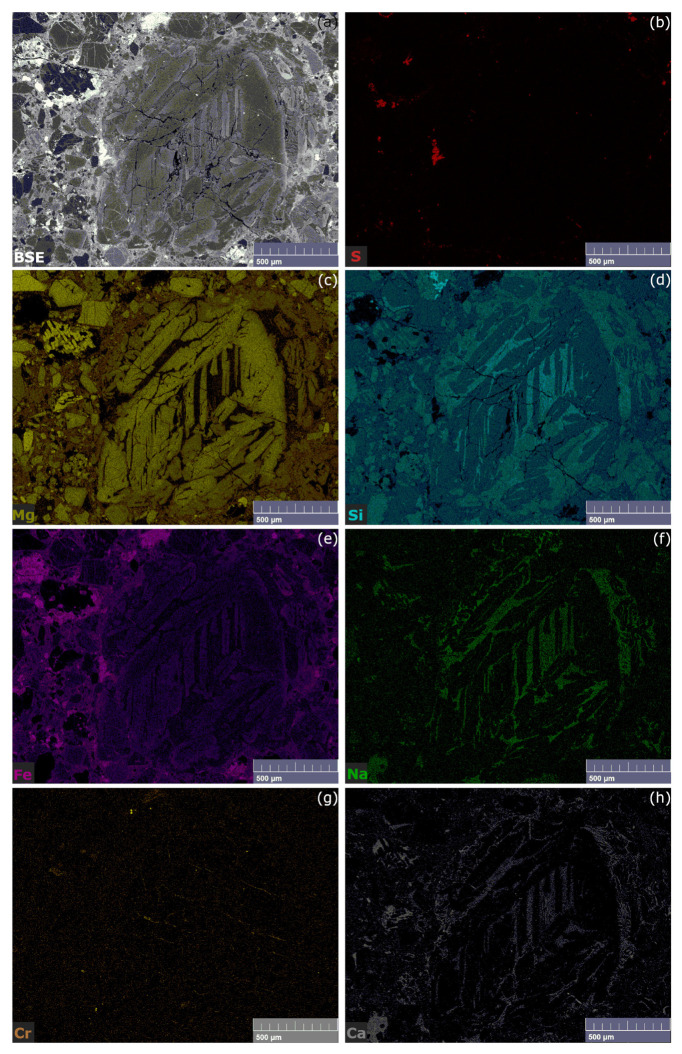
SEM-EDS results of chondrule B: (**a**) BSE image of the chondrule; (**b**–**h**) EM: (Red) S EDS map; (yellow) Mg EDS map; (blue) Si EDS map; (purple) Fe EDS map; (green) Na EDS map; (orange) Cr EDS map, (grey) Ca EDS map.

In Figure 6, the SEM-EDS and the relative EM results are displayed. As previously observed, by the BSE image (see Figure 6a) the differences between the concentrations of lower and higher Z have been reported; the same differences have been highlighted and deeply studied by the EM (see Figure 6b–h). In particular, the colour code is the same used for the mapping of chondrule A, adding only sulphur (see Figure 6b), which in this second case has been detected in appreciable concentrations. It must be noted the higher concentration of Fe is reported outside the chondrule, while the matrix between the forsterite and enstatite crystals resulted reach in Si and Na, as for Chondrule A.

## 4. Discussion

Based on the analyses performed, the two chondrules appeared to be composed by the same mineral phases, despite their different textures: chondrule A with porphyritic olivine and pyroxene, while the chondrule B presented barred olivine [25,33,34].

Forsterite, the magnesium rich phase of the olivine series, was disclosed in both chondrules, while the Raman analyses allowed to exclude a fayalite [Fe_2_SiO_4_] component, the iron rich phase of the same mineral series [35]. This fact has been confirmed by the cross application of the EDS analyses, both by spot and mapping tools, which highlighted for the olivine grains the high Mg concentration and Fe only in traces. Similarly, in both chondrules, the well-formed pyroxene crystals were made of enstatite mineral, again the magnesium rich phase of the series, and not of ferrosilite [Fe(SiO_3_)], the iron rich phase of the series. Again, this result has been confirmed by EDS spot and map investigations. The same behaviour has been observed also for diopside [CaMgSi₂O₆] vs. hedenbergite [CaFeSi₂O₆]: only the diopside has been detected. Forsterite, enstatite, and diopside resulted in the main Mg-silicate phases in the analysed chondrules: a segregation of the Fe during the chondrules formation process was evidenced.

Moreover, the absence of the ringwoodite phase could be correlated with a not extremely high metamorphic processes of the meteorite. We stress that through the use of micro-Raman mapping we could exclude its presence, an interesting result from our methodology point of view.

The advanced potentiality of the combined SEM-EDS and micro-Raman mapping has been highlighted also by the glass-feldspar-diopside detection: not only well crystallized minerals have been identified, but, thanks to the combination of the two mapping tools, the presence of crypto crystals of feldspar and diopside immersed in the matrix and crystallised near the rime of bigger enstatite and forsterite grains have been detected.

While in Martian meteorites anhydrite is not an extremely rare phase [36,37,38], in chondrite it is not reported as a typical mineral [5]. In the literature [7,25,39], the discussion about the presence of sulphur-reach phases in chondrite, such as troilite and oldhamite, has been reported. In particular, two main issues have been demonstrated in [32]: the first one is that none of the cubic monosulfide oldhamite, niningerite, or alabandite should present first-order Raman spectra, according to group theory, due to their ideal rock salt structure; while only the presence of elemental substitutions able to break the symmetry can produce broad Raman scattering; the second fact is the relative instability of those phases, with the risk of being damaged under the Raman laser beam. In the present work we could not identify the presence of possible monosulfides phases: the scattering is too broad to be highlighted by the mapping method and in a polyphasic sample some of those bands can be overlapped. We also hypothesized a correlation between the anhydrite discovery and a possible S-Ca phase alteration during the mapping analyses. Anyway, the presence of sulphur rich phases has been confirmed by the complementary EM.

Similar considerations are applicable to the iron oxides and their crystallinity. In fact, hematite Raman bands have been detected in both chondrules (Figure 2c,g and Figure 5c) in the corresponding Fe rich areas (Figure 3e and Figure 6e); nevertheless by the line spots Raman mapping, where the total exposure of the sample to the laser was less than the bi-dimensional map, only broad bands around 400 and 220 cm^−1^ have been observed (Figure 4 spots 3 and 4).

Thus, the iron oxides phases are confirmed, but an effect on their crystallinity correlated with the Raman’s source energy cannot be excluded [22,40].

By the combination of µ-RM and EM, the presence of a small grain of quartz in the area surrounding the Chondrule B has been detected. It is interesting to note that quartz is rarely found in meteorites and usually crystalline phases of Si are associated with tridymite and cristobalite or coesite, highlighting high pressure events occurring during the asteroid’s formation [21,41,42]. In the present work, none of those phases have been observed in the area where the quartz crystal has been identified. Moreover, on the basis of the Raman spectra, the quartz appeared well-crystallized, thanks to the faint presence of the moldavite characteristic Raman band at about 503 cm^−1^ and the sharp strong band at 465 cm^−1^ corresponding to the symmetric stretching of the Si-O bonds in the quartz structure [43,44]. To better explain the presence of quartz in this chondrite object, the statistics of the analysed chondrules must be increased as well as the information regarding the meteorite itself.

## 5. Conclusions

In the present work, the combined application of micro-Raman and SEM-EDS mapping has been tested, and proved to be simple and accurate for the determination of mineral phases at the micron size domain. A chondrite, which is a stony meteorite, has been used as a case study. We focused our attention on two chondrules: one with porphyritic texture of olivines and pyroxenes and the other one with barred olivines texture. A complete characterisation has been performed from a morphological, mineralogical, and chemical point of view. In fact, by the combination of micro-Raman and SEM mapping, not only on the same sample, but on the same portion of it, was the interpretation of the data using a complementary procedure allowed. Moreover, both techniques are spatially referred (mapping tool), and thanks to this fact, a double cross interpretation with the morphology and the relative grains position has been carried out.

Major crystalline phases, such as forsterite enstatite and diopside, have been identified by gathering their chemistry and their spectroscopic features; thus, the mineral phase identification with a strong iron segregation during the chondrule genesis has been confirmed. In addition, minor phases have also been detected, such as anhydrite, quartz, and hematite.

Finally, the double cross between the Elemental Mapping and µ-Raman Mapping of the matrix areas within the chondrule grains allowed the identification of the glass, pure in chondrule A, but with submicrometric crystals of feldspar in chondrule B.

The wide set of collected spectra and the potential of mapping tools have allowed an in-depth characterisation, also able to exclude the presence of specific mineralogical phases: this testifies the suitability of the combined methodology for meteorites studies, and will be helpful for meteorite characterisation and classification procedure, taking advantage of the most advanced techniques. Moreover, the identification of a single small quartz crystal in a very complex and polyphasic sample should be considered a good example of the methodology accuracy. It must also be noted that only non-destructive techniques have been applied, allowing to use a sample previously prepared, without requiring new material sampling. In fact, meteorites are unique objects, assimilable to Cultural Heritage items; thus, it is mandatory to develop new analytical methodologies, such as the one presented here, which do not require sample preparation or the application of destructive measurements. In this scenario, this study can be easily replicated to other meteorite samples, where a thin section is already available, and it is intended as part of a meteorite characterisation project, exploring new analytical protocols development in a non-destructive multianalytical and multidisciplinary approach.

## Data Availability

The raw/processed data required to reproduce these findings are available from the corresponding author on reasonable request.
